# Non-Uremic Calciphylaxis: An Unexpected Complication With Recombinant Human Parathyroid Hormone

**DOI:** 10.7759/cureus.15014

**Published:** 2021-05-13

**Authors:** Cory DeClue, Bhavana Chinnakotla, Michael J Gardner

**Affiliations:** 1 Internal Medicine, University of Missouri, Columbia, USA; 2 Diabetes and Endocrinology, University of Missouri, Columbia, USA

**Keywords:** non uremic calciphylaxis, recombinant pth, sodium thiosulfate, hypoparathyroidism

## Abstract

Calciphylaxis is a rare syndrome of calcific microvascular occlusion, whereas non-uremic calciphylaxis (NUC) is a subset of this disease in which renal impairment is not observed. Recombinant human parathyroid hormone (rhPTH) (1-84) is a medication approved for the management of hypocalcemia in patients with hypoparathyroidism. We present a case report of a 38-year-old woman with postoperative hypoparathyroidism treated with rhPTH who subsequently developed calciphylactic lesions on her abdomen. Multidisciplinary interventions included intravenous and intralesional sodium thiosulfate therapy, laboratory monitoring, dermatological wound care, and pain management. Calciphylaxis can rarely be precipitated by rhPTH due to its effect on calcium and phosphorus balance even in the setting of normal renal function. The use of calcium and calcitriol supplementation, complicated by factors such as female sex and obesity, may have contributed in this patient’s case. Hence, regular follow-up with tapering off of calcium and calcitriol supplementation is important in patients receiving rhPTH.

## Introduction

Calciphylaxis is a rare syndrome of microvascular occlusion in the subcutaneous adipose tissue and dermis, resulting in painful skin lesions and poor prognosis. Lesions are composed of calcium and phosphate, and elevated levels of either of these elements increase the risk of calciphylaxis [1]. While calciphylaxis is primarily associated with end stage renal disease (ESRD), rarely it has been identified in patients with normal renal function due to a variety of reasons, collectively called non-uremic calciphylaxis (NUC).

Even among patients with ESRD, calciphylaxis is quite uncommon, as evidenced by an estimated annual incidence rate of 0.04% in one registry of dialysis patients [2]. NUC is even rarer, accounting only for 5% of cases of calciphylaxis in a recent systematic review [3]. Another systematic review of NUC found that the most common risk factors were primary hyperparathyroidism, malignancy, warfarin use, and alcoholic liver disease [4]. It is interesting to note that the serum calcium and phosphate were normal in the majority of these cases reviewed.

Recombinant human parathyroid hormone (rhPTH) may be a rare cause of calciphylaxis. This is evident from two cases of NUC resulting from use of Teriparatide, a form of PTH consisting of the first 34 amino acids [5-6]. Two cases of Teriparatide-associated uremic calciphylaxis have also been described [7-8]. The confounding risk factors in these cases include two patients with glomerular filtration rates of 37 and 46 mL/min, while the other two patients had autoimmune disease treated with corticosteroids. Additionally, three of the patients were obese, and two of the patients were treated with warfarin therapy.

Natpara [rhPTH(1-84)] is an rhPTH injection approved as an adjunct to calcium and vitamin D to treat hypocalcemia in patients with hypoparathyroidism. It has been increasingly used by endocrinologists since its approval in 2015. Iatrogenic hyperparathyroidism resulting from rhPTH may cause devastating side effects. Here, we present a case of calciphylaxis in a woman treated with rhPTH(1-84).

## Case presentation

A 38-year-old female was diagnosed with primary hyperparathyroidism with an intact PTH (iPTH) of 389 pg/mL (normal range: 15-65 pg/mL) after she developed a renal calculus with serum calcium of 9.9 mg/dL (normal range: 8.6-10.2 mg/dL). Past medical history included hypertension, gestational diabetes mellitus, obesity with BMI 34, depression, chronic osteomyelitis of the left foot, and migraine disorder. Surgical history consisted of multiple foot surgeries and two cesarean sections. Family history included lung cancer in her father and hypertension in her mother. There was no history of tobacco use, and she drank two glasses of wine monthly. Medication list included vitamin D3 2000 IU/day, topiramate 75 mg/day, amlodipine 5 mg/day, fluvoxamine 50 mg/day, gabapentin 300 mg/day, and as needed clonazepam 0.5 mg.

Due to symptomatic hyperparathyroidism, she underwent parathyroidectomy of two out of four glands. Within days post-operatively, she developed symptomatic hypercalcemia of 12.8 mg/dL with an appropriately suppressed iPTH of 18.7 pg/mL. This was attributed to excess calcium supplementation, as extensive workup for hypercalcemia including repeat sestamibi scan, CT chest/abdomen, serum protein electrophoresis/urine protein electrophoresis (SPEP/UPEP), and bone marrow biopsy was unremarkable. She was started on cinacalcet 30 mg twice daily. After four doses, the patient developed muscle spasms and perioral paresthesia with hypocalcemia of 6.7 mg/dL despite calcitriol and calcium supplementation. Cinacalcet was stopped, and she was assumed to have postoperative hypoparathyroidism with an ionized calcium of 1.22 mmol/L (normal range: 1.15-1.25 mmol/L) and iPTH of 9.4 pg/mL two weeks after drug discontinuation. Serum calcidiol was 26 ng/mL (normal: 30-80 ng/mL) and 24-hour urine calcium was 327 mg (normal: 100-300 mg). rhPTH(1-84) 50 µg daily was started. Four months later, calcium was 9.2 mg/dL. Six months after this, her calcium was 7.6 mg/dL, phosphorus was 3.6 mg/dL (normal: 2.7-4.5 mg/dL), and iPTH was 12.6 pg/mL. She experienced muscle spasms, so rhPTH(1-84) was increased to 75 µg daily.

Seventeen months following initiation of rhPTH(1-84), the patient developed a painful right breast lesion that had free air on ultrasound. Emergent surgical excision was done, and pathology showed necrotic tissue with calcification consistent with calciphylaxis. At that time, serum calcium was 9.9 mg/dL, phosphorus was 3.3 mg/dL, and iPTH was 44 pg/dL. Over the next two months, bilateral breast lesions erupted that required mastectomies. Following this, calcitriol was lowered to 0.5 µg twice daily, but rhPTH(1-84) was continued.

Two months later, she developed a new abdominal calciphylaxis lesion and presented to our center. Her lab results at admission, during hospital course, and at three-month post-discharge are illustrated in Table 1.

**Table 1:** Laboratory values for the patient while on Natpara, off Natpara, and at three months post-discharge.

Lab Values	At admission (On Natpara)	During hospitalization (Off Natpara)	Three-month post-discharge	Reference range
Calcium (mg/dL)	9.5	8.2	9.2	8.6-10.2
Phosphorus (mg/dL)	2.9	2.5	4	2.7-4.5
Calcium-Phosphorus product (mg/dL)	27.55	20.5	36.8	
Ionized Parathyroid Hormone (pg/dL)	67.9	66.7	69.7	15-65
Sodium (mmol/L)	137	139	139	136-145
Potassium (mmol/L)	3.5	3.4	4.3	3.5-5.2
Chloride (mmol/L)	96	105	102	98-107
Carbon Dioxide (mmol/L)	24	23	25	22-29
Anion gap (mmol/L)	17	10	12	0-20
Blood Urea Nitrogen (mg/dL)	17	6	10	6-20.0
Creatinine (mg/dL)	0.73	0.47	0.61	0.5-1.2
Albumin (g/dL)	4.1	3.3	4.2	3.5-5.1
Vitamin D (ng/mL)		31.6	36	30-80
White Blood Cells (x10^9^/L)	15.1	9.2	13.7	3.5-10.5
Platelets (x10^9^/L)	464	409	394	150-450
Glucose (mg/dL)	87	99	104	74-106

**Figure 1:**
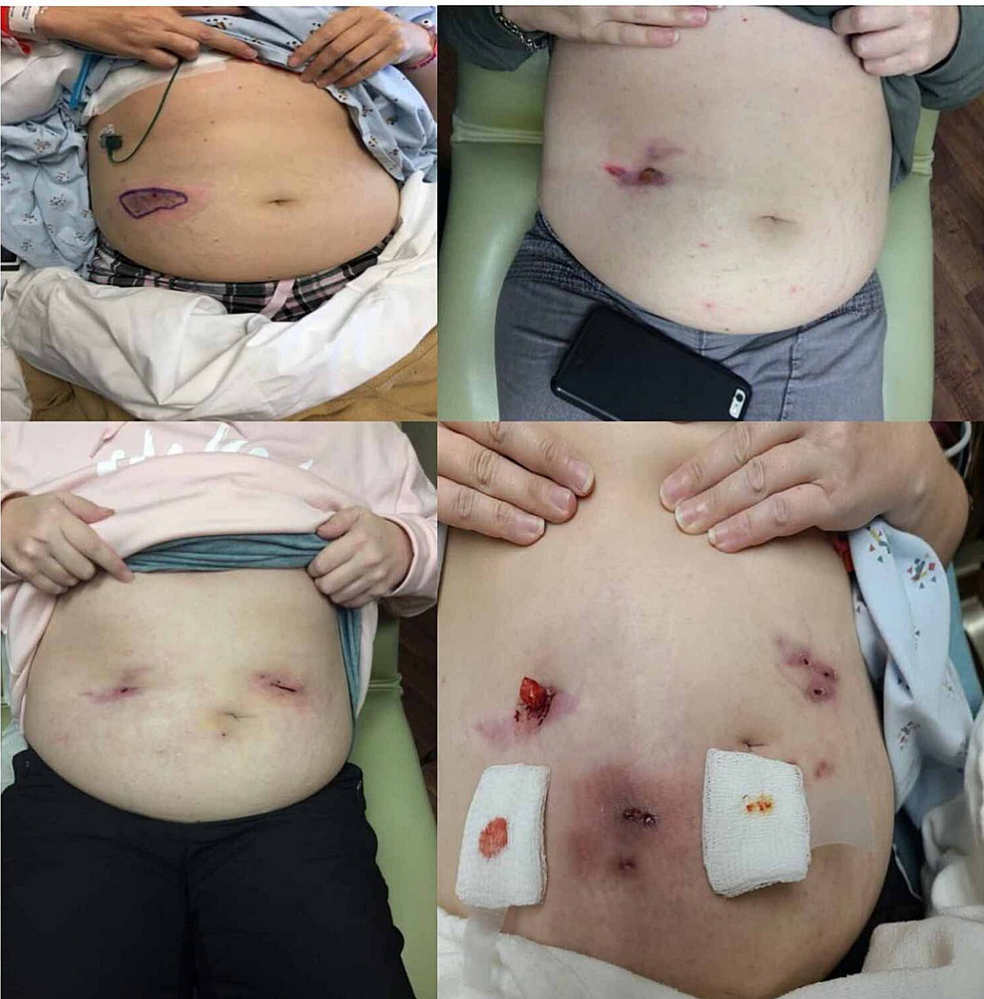
The calciphylaxis lesions over time. Lesions shown are at admission (top left), six months later (top right), seven months later (bottom left), and ten months later (bottom right).

As noted in the table, renal function testing remained normal. Upon withholding the rhPTH(1-84), iPTH remained as high as 70 pg/mL, questioning her diagnosis of hypoparathyroidism. Calcitriol, calcium supplements, and rhPTH(1-84) were discontinued. A multidisciplinary treatment plan with sodium thiosulfate (STS) 25 g/100 mL IV three times per week along with pain management and wound care was initiated. The patient’s calcium and phosphorus remained normal throughout her course (Table 1). Her wounds progressed well on the STS infusions for five months (Figure 1), but due to incomplete closure, she was seen in dermatology clinic for intralesional STS injections.

She has continued to have new abdominal lesions despite discontinuation of Natpara for over one year. Due to significant pain, the patient continues to follow with palliative care clinic for opioid management.

## Discussion

The pathogenesis of calciphylaxis is uncertain, despite growing work in this field as of late. Current theory suggests that local microvascular calcification leads to chronic ischemia, which then causes infarction via endothelial injury and microthrombus formation. This initial step appears to be due to a cell-mediated process involving a deficiency of calcification inhibitors. Matrix Gla protein (MGP) is an extracellular matrix protein that strongly inhibits calcification when carboxylated. This carboxylation step is dependent on vitamin K, leading many to speculate that this is the reason warfarin is a risk factor for calciphylaxis [9]. Another implicated inhibitor is fetuin-A, which leads to formation of calciprotein particles that help transport minerals through the body. Fetuin-A has been found to be downregulated in chronic inflammatory diseases, which may explain the increased calciphylaxis risk in people with uremia and autoimmune diseases [10].

In addition to a lack of calcification inhibition, there seems to be an increased pro-calcification response in calciphylaxis. Adipocytes have been shown to secrete cytokines that can lead to differentiation of vascular smooth muscle cells into procalcific cells, and this is one mechanism through which STS prevents calcification [11]. Additionally, this provocation of differentiation is stimulated by hyperphosphatemia, hypercalcemia, and hyperglycemia, explaining the increased risk in hyperparathyroidism and diabetes [12-13]. Despite all of these discoveries, there is still much to be learned about how and why calciphylaxis occurs.

Our patient developed iatrogenic hyperparathyroidism from rhPTH(1-84) in the setting of intact parathyroid gland function. The calcium, calcitriol, and vitamin D supplements most likely contributed to imbalance in the calcium and phosphorous metabolism resulting in this devastating disease. Hence, careful diagnosis and patient selection are important prior to the initiation of rhPTH(1-84) therapy.

Unlike the advancements in understanding the pathogenesis of calciphylaxis, treating this disease has not been as fruitful. Due to its complex nature, a multidisciplinary approach involving nephrologists, endocrinologists, plastic surgeons, dieticians, and wound care clinicians is paramount to success. In uremic calciphylaxis, elevated serum calcium and phosphate should be treated by increasing hemodialysis frequency and reducing intake [14]. Additionally, calcium-based phosphate binders should be avoided, and vitamin D supplements should be discontinued. Cinacalcet can be used for PTH suppression, and warfarin should be discontinued [15]. Bisphosphonates have been shown to improve pain and severity of skin lesions in retrospective studies but require controlled trials to determine true efficacy [16].

In addition to reducing vascular calcification, concurrent decalcification of vessels is also needed. STS has become the standard therapy due to the water-soluble thiosulfate chelation of calcium from the vessel wall, as well as antioxidant properties to reduce inflammation and thrombosis. In a recent meta-analysis of STS use in uremic calciphylaxis, improvement in lesions was observed in 70.1% of patients [17]. Another agent being used for treatment is vitamin K, which has been shown to reduce vascular calcification in animal models [18]. A newer agent, called SNF472 (intravenous myo-inositol hexaphosphate), blocks the formation of hydroxyapatite and has been assessed recently for treatment in calciphylaxis [19]. Finally, a change in medium such as double-filtration apheresis and hyperbaric oxygen therapy has reported some use in STS-refractory calciphylaxis cases.

As noted from the above discussion, most of the experience in diagnosing and treating calciphylaxis is evident from ESRD patients, while non-uremic calciphylaxis remains to be explored. Given the rarity of this phenomenon, there is currently a national registry for calciphylaxis which may be a potential tool for exploring its etiology, risk factors, and management. As we await more robust studies in NUC, we as endocrinologists must be aware of hyperparathyroidism, both primary and iatrogenic from rhPTH(1-84), as a risk given the associated morbidity and mortality.

## Conclusions

Our case highlights the need to monitor hypoparathyroid patients treated with rhPTH and calcitriol for calciphylaxis. Calcium and calcitriol supplementation should be adjusted, and patients should be closely followed to monitor for endogenous parathyroid function. The majority of calciphylaxis occurs in the setting of renal impairment, notably in dialysis patients. However, there can be a subset with normal renal function. Calciphylaxis, regardless of the renal function, still has poor outcomes. Gaining a better understanding of the precipitating factors for this disease process not only can establish effective therapies but can also lead to better treatment of calcium disorders when concomitantly present.
